# Integration of Chronic Oncology Services in Noncommunicable Disease Clinic in Rural Rwanda

**DOI:** 10.5334/aogh.2697

**Published:** 2020-03-23

**Authors:** Robert Rutayisire, Francis Mutabazi, Alice Bayingana, Ann C. Miller, Neil Gupta, Gedeon Ngoga, Eric Ngabireyimana, Ryan Borg, Emmanuel Rusingiza, Charlotte Bavuma, Bosco Bigirimana, Fulgence Nkikabahizi, Marie Aimee Muhimpundu, Gene Bukhman, Paul H. Park

**Affiliations:** 1 College of Medicine and Health Sciences, University of Rwanda, Kigali, RW; 2 Ministry of Health, Kigali, RW; 3 Partners In Health/Inshuti Mu Buzima, Kigali, RW; 4 Department of Global Health and Social Medicine, Harvard Medical School, Boston, US; 5 Division of Global Health Equity, Brigham and Women’s Hospital, Boston, US; 6 University Teaching Hospital of Kigali, Kigali, RW; 7 Non-Communicable Disease Division, Rwanda Biomedical Center, Kigali, RW

## Abstract

**Background:**

In rural sub-Saharan Africa, access to care for severe non-communicable diseases (NCDs) is limited due to myriad delivery challenges. We describe the implementation, patient characteristics, and retention rate of an integrated NCD clinic inclusive of cancer services at a district hospital in rural Rwanda.

**Methods:**

In 2006, the Rwandan Ministry of Health at Rwinkwavu District Hospital (RDH) and Partners In Health established an integrated NCD clinic focused on nurse-led care of severe NCDs, within a single delivery platform. Implementation modifications were made in 2011 to include cancer services. For this descriptive study, we abstracted medical record data for 15 months after first clinic visit for all patients who enrolled in the NCD clinic between 1 July 2012 and 30 June 2014. We report descriptive statistics of patient characteristics and retention.

**Results:**

Three hundred forty-seven patients enrolled during the study period: oncology – 71.8%, hypertension – 10.4%, heart failure – 11.0%, diabetes – 5.5%, and chronic respiratory disease (CRD) – 1.4%. Twelve-month retention rates were: oncology – 81.6%, CRD – 60.0%, hypertension – 75.0%, diabetes – 73.7%, and heart failure – 47.4%.

**Conclusions:**

The integrated NCD clinic filled a gap in accessible care for severe NCDs, including cancer, at rural district hospitals. This novel approach has illustrated good retention rates.

## Introduction

In sub-Saharan Africa (SSA), non-communicable diseases (NCDs) account for more than a quarter (28%) of the disease burden by DALYs [1]. This disease burden can further be broken down to the more common, less complex NCDs (e.g., uncomplicated hypertension, asthma, type 2 diabetes) as well as the more severe conditions, such as malignancies, type 1 diabetes, and heart failure. Early detection and management are of critical importance, especially for more severe NCDs, and can help prevent both acute complications and/or early mortality. In SSA, heart failure represents 0.75% of total DALYs, and neoplasms is 4.7% [1]. Given the expertise and resources readily available at many referral and university teaching hospitals, a more vertical disease-specific, top-down approach (for individual diseases, including each NCD) is quite common in urban centers [2]. Such an approach with appropriate resources allows for long-term follow-up of both common and severe NCDs. At primary health centers service delivery models for common NCDs do exist, however, there is little documented experience with integrated care teams that address severe NCDs at district hospital outpatient clinics [3].

The World Health Organization (WHO) recommends strengthening primary care in both urban and rural settings as a critical pathway towards achieving universal health coverage [4]. Specifically, the UN Sustainable Development Goals (SDGs) includes targets calling for universal health coverage for all diseases, including NCDs. With effective scale-up of NCD services, the UN is seeking a one third reduction in premature mortality from NCDs through prevention and treatment by 2030 [5]. With 62% of sub-Saharan Africa (SSA) living in a rural setting [6], establishing such effective coverage in the region would require establishment of accessible services at the district hospital level in both urban and rural areas. However, the delivery of NCD care in rural district hospitals has significant challenges due to limited infrastructural resources, trained medical personnel, and other critical factors [4]. With such limitations, the reliance on a vertical approach can lead to inefficient use of precious staff and equipment, rendering the facility incapable of meeting the broader population needs surrounding NCDs and other common diseases [7]. Furthermore, vertical programs can be burdensome for patients with comorbidities due to gaps in continuity of care and increased time and costs spent seeking care in multiple clinics [8].

Rwanda is one of the few low-income countries in SSA that has worked to decentralize care for severe chronic NCDs to the level of district hospitals nationally. As opposed to vertical, single disease-based clinics, a more integrated approach was utilized. This decentralization strategy began in 2006, when the Rwanda Ministry of Health (RMoH), in collaboration with Partners In Health/Inshuti Mu Buzima (PIH/IMB-an international non-governmental organization), initiated an integrated district-level program at Rwinkwavu District Hospital (RDH). This program would ultimately focus on the management of both severe and less complex chronic NCDs: heart failure, advanced hypertension, type 1 and 2 diabetes, chronic respiratory disease (CRD), as well as end-stage liver and kidney disease in a single resource-efficient delivery platform [9]. This nurse-led clinic provides initial diagnosis, long-term follow-up, and patient education through adaptation of carefully selected evidence-based interventions appropriate for the resource-limited setting.

Before 2011 in Rwanda, oncology diagnosis and treatment were limited to referral hospitals for only a few types of cancers, and costs were largely prohibitive to the majority of the population [10]. Due to the critical need for cancer care, which at the time was restricted to referral centers, oncology inpatient and outpatient services were initiated at RDH in 2006 for a few patients across a narrow spectrum of cancers, including Hodgkin lymphoma and acute lymphoblastic leukemia. After gaining more experience and expanding the breadth of diseases, the RDH leadership elected to add the outpatient oncology services into the integrated NCD clinic in 2011. The oncology services and the subsidization of costs were supported by PIH/IMB, Dana-Farber/Brigham & Women’s Cancer Center, and other partners. The addition of specialized outpatient cancer services, inclusive of diagnosis, treatment, and palliative care, was a novel alteration to the integrated NCD clinic approach, and therefore required unique implementation practices and patient management components that were particularly challenging at a rural district hospital. Although by 2015 most curative services had been transferred from RDH to a newly established and PIH-supported national cancer center located in the Northern Burera District, the initial evaluation and chronic follow-up for oncology patients within the hospital catchment area remained within the integrated NCD clinic at RDH.

The purpose of this initial study is to describe the early RDH experiences with integration of care for severe chronic NCDs including malignancies. We describe clinic staffing, initial training, organization, as well as patient demographics at enrollment, and 12-month patient outcomes. The planning and operational impact of adding cancer services are also described.

## Materials and Methods

### Study setting and implementation process

**Figure 1:**
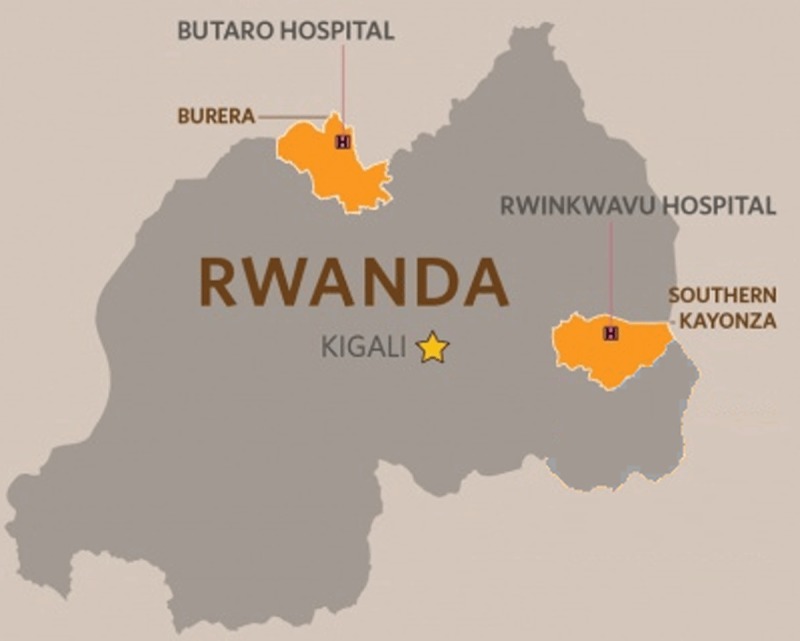
Map of Rwanda with catchment area of Rwinkwavu District Hospital (Southern Kayonza District) highlighted along with the location of the Butaro Cancer Center of Excellence, the primary referral hospital for cancer service.

This study was conducted at RDH, a hospital located in Southern Kayonza District in the Eastern Province of Rwanda (see Figure 1). The hospital’s catchment area covers a population of approximately 179,000 and encompasses eight health centers. The population of Southern Kayonza is predominantly rural (90%), female (51.6%) and young, with 49.4% of the population less than 18 years of age in the 2012 census [11]. In addition to the NCD clinic, RDH houses neonatal, pediatric, maternal, internal medicine, surgical, laboratory, radiology, pharmacy and general outpatient departments.

The initial step in the integrated NCD clinic’s implementation focused on governance [12]. PIH’s strong partnership with the RMoH at both the central and district levels allowed for collective strategic planning and commitment. Subsequent steps included drafting and approving national NCD clinical and operational protocols for heart failure, hypertension, type 1 and 2 diabetes, chronic kidney disease, and palliative care. Based on the protocols, training curricula for nurses and physicians helped establish a new cadre of Rwandan health care delivery pioneers for management of severe chronic NCDs. Using a combination of practical and didactic training, in the first year (2007–2008) four nurses from outpatient services and six general practitioners from inpatient services were trained in multiple areas of NCD care delivery, such as diagnostics and monitoring, which included the following specialized tools: echocardiography, International Normalized Ratio (INR), hemoglobin A_1c_ (HbA1c), and peak flow meter. The duration of the combined didactic and practical training was two months. Trainers included US-based internal medicine, cardiology, and pulmonology specialists. Additionally, gaps in the availability of essential medicines and equipment were identified and addressed. Initial procurement operations were heavily supported and subsidized by PIH/IMB; these supports and subsidies have since been substantially reduced. The integrated NCD clinic was housed in a single room with a desk, filing cabinet for patient medical records, a laptop computer, one exam table, and one of each of the following pieces of medical equipment: ultrasound with cardiac probe, stethoscope, sphygmomanometer, peak flow meter, Hb_A1c_ point-of-care machine, INR point-of-care machine, monofilament, glucometer, and a weight scale.

Disease-specific clinical forms were developed with input from both clinicians and monitoring and evaluation (M&E) specialists; training on completion of forms was also provided. These forms were later programmed into PIH/IMB’s electronic medical record (EMR) system, OpenMRS (OpenMRS Inc., Indianapolis, USA), to allow for continuity of care and more robust M&E and research. To support continuity and comprehensive care, criteria were developed to guide social workers in identifying vulnerable patients who required social assistance in the form of subsidizing transportation fees and distributing food packages.

Essential human resources within the integrated NCD clinic included three nurses, a general practitioner, a data officer and a clerk. NCD-trained nurses led the majority of patient encounters. The general practitioner attended to more complex cases such as patients not responding to standardized treatment regimens or those at especially high risk for clinical exacerbation. The data officer managed patient data and files, transcribed data from the paper forms into the EMR, and monitored form completeness. The clerk facilitated administrative workflow within the clinic and its interactions with the rest of the hospital. In addition, a cardiologist and an endocrinologist visited the clinic on a monthly basis to evaluate complex patients as well as newly diagnosed patients. These specialists also provided direct mentorship and education to the clinic staff.

The integrated NCD clinic operated on a weekly schedule with each day of the week exclusively dedicated to a particular disease unless the patient had multiple NCD diagnoses, in which case s/he would receive treatment for all illnesses during one clinic visit. Patients were referred to the NCD clinic from health centers, the RDH’s general outpatient department, inpatient departments, self-referral, or other district hospitals without such specialized services. Ultimately, the clinic was able to establish and communicate clear referral criteria for complex cases requiring a higher level of care.

The addition of oncology services to the integrated NCD clinic provided an opportunity to establish a model integrated district-level approach that provided decentralized cancer care with referrals to higher levels of care as needed. Given the unique intricacies of cancer care delivery, program leadership developed and implemented new training and referral pathways for both diagnosis and treatment. A two-month national oncology training inclusive of both didactic and practical components was organized at RDH in 2013 and covered both inpatient and outpatient content. Training of physicians and subsequent care delivery at RDH was focused on initial evaluation, including peripheral mass and bone marrow biopsy, staging, and initial treatment plan for select cancers, including lymphoma, leukemia, breast, and cervical cancers. For treatment, RDH provided a limited selection of intravenous chemotherapy and oral hormonal treatment regimens as well as palliative care, however the intravenous chemotherapy would later be phased out. Referral facilities were routinely used for more invasive biopsy, pathology diagnosis, advanced radiography, advanced intravenous chemotherapy, surgery, and radiotherapy. The primary referral facility was Butaro Cancer Center of Excellence (BCCOE), in Butaro Hospital, Northern Province. Central University Hospital in Kigali (CHUK), Rwanda Military Hospital, also in the capital city, Kigali, and Central University Hospital in Butare (CHUB) of the Southern Province provided key support as referral hospitals, especially with respect to treatment plan, follow-up, surgeries, CT scans, and biopsies [13]. Radiotherapy required transfer to cancer centers in Uganda and Kenya [14].

Regarding training and mentorship, US-based oncologists and nurses from Dana-Farber/Brigham & Women’s Cancer Center supported the RMoH in clinical protocol writing, on-site training, routine conference calls and email communication surrounding specific patient cases and programmatic challenges. Drug procurement was also subsidized by external partners. Advanced medications, including chemotherapy and warfarin, were not readily available through the public supply chain, so PIH procured these medications as to support the RMoH until the public sector was able to procure independently. The RMoH and partners also established an oncology platform within the OpenMRS EMR.

### Study design and population

This was a retrospective cohort study of new patients who enrolled in the RDH integrated NCD clinic with the addition of oncology services, between 1 July 2012 to 30 June 2014. All qualifying patients were then assessed for 12 months as described below. Patients of all ages diagnosed with heart failure, diabetes, CRD, hypertension or a type of cancer were included in the analysis.

### Data collection and analysis

De-identified data on demographic, clinical and outcome variables were extracted from the EMR. As some diseases did not have representative electronic forms, EMR records were not available for patients with chronic kidney and liver disease and, thus, those patients were not included in this study. If a patient was enrolled in more than one disease program, each enrollment was treated as a separate record. Data were reported by disease group to better describe the clinical profile of patients, and the most relevant characteristics of each disease group (e.g. diabetes type, cancer type, heart failure diagnosis) were reported if those data was available. The primary outcome was patient status at 12 months after his/her enrollment date. A patient was considered to be “alive and in care” if s/he had a recorded follow-up visit within ±3 months of the 12-month milestone; “lost to follow-up” (LTFU) if s/he did not have a recorded visit within ±3 months of the 12-month milestone; and “dead” if s/he had a date of death recorded in the EMR during the study window. Extracted data were cleaned and analyzed using Stata v14 (College Park, TX). Descriptive statistics were calculated and number and percentages presented for categorical data.

### Ethical Approval

The research protocol was approved by the Rwanda National Ethics Committee, National Health Research Committee, and by the Brigham and Women’s Hospital Institutional Review Board in Boston, Massachusetts, USA, prior to the initiation of the study.

## Results

Overall, 347 new patients were enrolled in the integrated NCD clinic at RDH between 1 July 2012 and 30 June 2014, of which 61.6% (n = 214) were from outside the RDH catchment area. 74.1% (n = 258) of the patients were female (Table 1). The distribution among the five primary diagnosis groups was: cancers (71.8%, n = 249) heart failure (11.0%, n = 38), diabetes (5.5%, n = 19), hypertension (10.4%, n = 36), and chronic respiratory disease (1.4%, n = 5). Of the 347 patients, 44.4% (n = 154) were between 40–59 years. Only 25.3% (n = 63) of oncology patients resided in the RDH catchment area, in comparison to 71.4% (n = 70) of non-oncology patients. Of 133 patients who resided within RDH’s catchment area, 74.4% were female. The distribution among five primary diagnosis groups for those from RDH’s catchment area was: cancers (47.4%, n = 63), heart failure (18.8%, n = 25), diabetes (10.5%, n = 14), hypertension (21.1%, n = 28), and chronic respiratory diseases (2.3%, n = 3).

**Table 1:** Demographic characteristics of patients enrolled in RDH integrated NCD clinic by primary diagnosis (2012–2014).

	Diabetes (N = 19)		Hypertension (N = 36)		Heart failure(N = 38)		CRD (N = 5)		Oncology (N = 249)		Total (N = 347)	

	n	%	n	%	n	%	n	%	n	%	N	%

**Age**												
0–19	3	15.8	0	0.0	14	36.8	2	40.0	27	10.8	46	13.3
20–39	6	31.6	6	16.7	5	13.2	1	20.0	65	26.1	83	23.9
40–59	5	26.3	19	52.8	10	26.3	1	20.0	119	47.8	154	44.4
60+	5	26.3	11	30.6	9	23.7	1	20.0	38	15.3	64	18.4
**Sex**												
Male	9	47.4	10	27.8	6	15.8	1	20.0	63	25.3	89	25.6
Female	10	52.6	26	72.2	32	84.2	4	80.0	186	74.7	258	74.4
**RDH catchment Resident**												
No	5	26.3	8	22.2	13	34.2	2	40.0	186	74.7	214	61.7
Yes	14	73.6	28	77.8	25	65.8	3	60.0	63	25.3	133	38.3

**Table 1a:** Demographic characteristics of patients enrolled in RDH integrated NCD clinic by primary diagnosis in RDH catchment area (2012–2014).

	Diabetes (N = 14)		Hypertension (N = 28)		Heart failure (N = 25)		CRD (N = 3)		Oncology (N = 63)		Total	

	n	%	n	%	n	%	n	%	n	%	n	%

**Age (n = 133)**												
0–19	2	14.3	0	0.0	8	32.0	2	66.7	8	12.7	20	15.0
20–39	4	28.6	5	17.9	4	16.0	0	0.0	17	27.0	30	22.6
40–59	3	21.4	14	50.0	6	24.0	0	0.0	28	44.4	51	38.4
60+	5	35.7	9	32.1	7	28.0	1	33.3	10	15.9	32	24.1
**Sex (n = 133)**												
Male	6	42.9	7	25.0	4	16.0	0	0.0	23	36.5	40	30.1
Female	8	57.1	21	75.0	21	84.0	3	100.0	40	63.5	93	69.9

Table 2 describes specific primary diagnoses at baseline, separately for all patients and RDH catchment patients. Of the 19 total patients enrolled in the diabetes program, 21% (n = 4) were diagnosed with Type 1 diabetes, 52.6% (n = 10) were diagnosed with Type 2 diabetes, and 26.3% (n = 5) had no type recorded in the EMR (Table 2). Of the 38 patients with heart failure, 21.9% (n = 9) were diagnosed with cardiomyopathy, 23.7% (n = 5) with congenital heart disease, 13.2% (n = 4) with rheumatic heart disease, 10.5% (n = 4) with hypertensive heart disease, and 42.1% (n = 16) had no specified type of heart failure indicated. Of the 249 patients diagnosed with a type of cancer, the three most commonly diagnosed cancers were: breast cancer (19.3%, n = 48), gynecological cancer (cervical cancer, endometrial cancer, molar pregnancy, choriocarcinoma, and vulva cancer; 19.3%, n = 48), and leukemia (chronic myelogenous leukemia [CML], chronic lymphocystic leukemia, and acute lymphoblastic leukemia; 7.6%, n = 19). RDH catchment area patients were distributed similarly.

**Table 2:** Types of primary diagnoses of patients enrolled in RDH integrated NCD clinic (2012–2014).

	All patients		Catchment area only	

	n	%	n	%

**Diabetes types (N = 19)**			**N = 14**	
Type 1 diabetes	4	21	2	14.3
Type 2 diabetes	10	52.6	7	50.0
Type not reported	5	26.3	5	35.7
**Heart failure types (N = 38)**			**N = 22**	
Rheumatic heart disease	4	10.5	3	13.6
Congenital heart disease	5	13.2	3	13.6
Hypertensive heart disease	4	10.5	2	9.1
Cardiomyopathy	9	23.7	7	31.8
Type not reported	16	42.1	7	31.8
**Cancer (N = 249)**			**N = 62**	
Breast cancer	48	19.3	7	11.3
Gynecological cancer	48	19.3	14	22.6
Cervical cancer	41	85.4	13	92.9
Endometrial cancer	4	8.3	1	7.1
Molar pregnancy	1	2.1	0	0
Choriocarcinoma	1	2.1	0	0
Vulva cancer	1	2.1	0	0
Leukemia	19	7.6	4	6.5
Chronic myelogenous leukemia	17	89.5	2	50
Chronic lymphocytic leukemia	1	5.3	1	25
Acute lymphoblastic leukemia	1	5.3	1	25
Other Cancers	134	53.8	37	59.7
Thyroid cancer	3	2.2	2	5.4
Male reproductive cancer	4	3	1	2.7
Skin cancer	8	6	4	10.8
Sarcomas	3	2.2	2	5.4
Multiple myeloma	2	1.5	0	0
Lung cancer	1	0.7	1	2.7
Gastrointestinal cancer	6	4.5	0	0
Head and neck cancer	4	3	1	2.7
Lymphoma	6	4.5	3	8.1
Other (not specified)	97	72.4	23	62.2

After 12 months of follow-up, overall, 76.4% (n = 266) of patients were known to be alive and in care, 7.2% (n = 25) had died, and 10.1% (n = 35) were lost to follow-up (Table 3). Retention in care varied by primary diagnosis. Oncology had the highest proportion of patients retained in care at 12 months (81.6%, n = 204), followed by patients with hypertension (75.0%, n = 27), diabetes (73.7%, n = 14), chronic respiratory disease (CRD) (60.0%, n = 3), and heart failure (47.4%, n = 18). Patients with CRD had the highest proportion of deaths during the time period (20.0%, n = 1), followed by oncology (9.2%, n = 23) and heart failure (2.6%, n = 1). Among the 133 patients from the RDH catchment area, after 12 months of follow-up 73.7% (n = 98) of patients were known to be alive and in care, 3% (n = 4) had died, 5.3% (n = 7) were in remission from cancer, and 18.1% (n = 24) were lost to follow-up (Table 3a). Of patients from the RDH catchment area, oncology also had the highest proportion of patients retained in care at 12 months (84.1%, n = 53), followed by patients with diabetes (78.6%, n = 11), hypertension (71.4%, n = 20), heart failure (52.0%, n = 13), and CRD (33.3%, n = 1). No patients with diabetes or hypertension were known to have died; of the four RDH catchment area residents that died, two were oncology patients, one was a heart failure patient and one was a CRD patient.

## Discussion

Limited documentation exists surrounding the role of district hospital outpatient services in the management of oncologic disease, which has largely been restricted to referral centers at the regional or national level. Previously we have described program outcomes related to heart failure, diabetes, and CRD [15,16,17]. Here, we examine the integration of patients with malignancies as part of this service. To the best of our knowledge, this is the first description of integration of chronic care for malignancies at a district level hospital in a low-income SSA setting.

During the period of this study, 71% of the patients with oncologic disease presented from outside of the hospital catchment area (see Tables 1 and 1a). Effectively, the hospital was serving as a regional cancer referral center prior to the strengthening of cancer care at other central facilities. However, even when just considering the patients in the district hospital’s catchment area, oncology patients still made up a substantial proportion (47.3%) of severe chronic NCD patients. Given the projected prevalence of the various NCDs [18,19], our study exhibited an overrepresentation of malignancies in comparison to other severe chronic NCDs. It is possible that case finding for malignancies may be high relative to other severe chronic NCDs because of the more overt presentation (e.g., palpable mass) for many solid tumor cancers. Furthermore, we noted a greater proportion of patients within the catchment area being of older age. This is likely due to the challenges faced by the elderly for longer transportation.

We note a high proportion (74%) of women in our patient population. This is likely due to the projected high prevalence of women’s cancers in SSA [1]. According the Global Burden of Disease study, approximately 65% of prevalent cancer cases in SSA are in women, as well as 57% of RHD cases and 51% of heart failure more generally. Additionally, the REMEDY registry of heart failure in low-income countries had a greater female-predominance (66%), also affected likely by greater interaction with the health system among women [20].

In the short term, retention of patients has been high in comparison to chronic disease studies in similar settings. We speculate that there may be several possible factors that are responsible for these high levels of retention. First, decentralized approaches to chronic care at the district level reduces the barriers of travel distance and cost to the patient [21]. However, there was no clear trend in retention when comparing patients living outside versus inside the catchment area (see Tables 3 and 3a). Second, the integrated NCD clinic tracks patients who miss clinic visits on a case-by-case basis via phone calls and home visits. Additionally, regular clinic days for specific conditions combined with the ability of staff to manage multiple diagnoses allow patients to be treated at one place at one time, instead of having to go to multiple sites at multiple times. Finally, the cost of care to the patient was substantially subsidized. Food packages were provided to the lowest economic groups, and in many cases financial assistance was provided to support travel to the facilities. Given that district hospitals are still quite far from where most patient live, we believe that this kind of social protection is important to prevent impoverishment and increase access for the poorest patients with severe chronic conditions. Community health workers (CHWs) were not routinely utilized per a fixed protocol. However, some of the more vulnerable patients were assigned CHWs to support home-based care and monitoring as well as adherence to appointments. More structured application of CHWs to the NCD program can enhance the retention to care.

**Table 3:** Patient status 12 months after enrollment date into RDH integrated NCD clinic by primary diagnosis (2012–2014).

	Diabetes N = 19		Hypertension N = 36		Heart failure N = 38		CRD N = 5		Oncology N = 249		Total N = 347	

	n	%	n	%	n	%	n	%	n	%	N	%

**Outcomes**												
Alive in care	14	73.7	27	75.0	18	47.4	3	60.0	204	81.6	266	76.6
Died	0	0.0	0	0.0	1	2.6	1	20.0	23	9.2	25	7.2
In remission	0	0.0	0	0.0	0	0.0	0	0.0	21	8.8	21	6.1
Lost to follow up	5	26.3	9	25.0	19	50.0	1	20.0	1	0.4	35	10.1

**Table 3a:** Patient status 12 months after enrollment date into RDH integrated NCD clinic by primary diagnosis in RDH catchment area (2012–2014).

	Diabetes N = 14		Hypertension N = 28		Heart failure N = 25		CRD N = 3		Oncology N = 63		Total N = 133	

	n	%	n	%	n	%	n	%	n	%	n	%

**Outcomes**												
Alive in care	11	78.6	20	71.4	13	52.0	1	33.3	53	84.1	98	73.7
Died	0	0.0	0	0.0	1	4.0	1	33.3	2	3.2	4	3.0
In remission	0	0.0	0	0.0	0	0.0	0	0.0	7	11.1	7	5.3
Lost to follow up	3	21.4	8	28.6	11	44.0	1	33.3	1	1.6	24	18.1

For the majority of curable oncology cases, management at district-level hospitals requires access to pathology services and treatment support from referral facilitates. We believe, based on our experiences that it appears to be feasible to manage stable patients at first level hospitals once diagnosis and therapeutic support has been established at a tertiary facility. Some cancers, such as early-stage estrogen receptor-positive breast cancer and CML, may only require oral therapy and be treated entirely at a district hospital [20]. For palliative care patients, including pain control, the management of symptoms such as nausea and provision of psychosocial support can be delivered in an integrated, decentralized fashion. Nevertheless, most of the cases required referral to Butaro with the exception of a few early stage, hormone receptor sensitive breast cancer cases and CML. RDH was responsible for the initial evaluation and diagnosis, and in some cases staging and then follow-up after completion of treatment. In future studies, it would be of value to include specific proportions of patients who required the various referral services.

The inclusion of oncology into the integrated NCD platform not only altered the patient volume landscape, but required several additional implementation steps surrounding training, mentorship, and referral patterns. There were two phases of care for oncologic patients. The first phase was establishing the diagnosis and treatment plans, which was primarily driven by generalist physicians [23]. This phase also required training and mentorship of RDH providers for specialized skills, such as biopsy and interpretation of pathology results. The second phase of care has shifted part of the responsibility of care to nurses. Specifically, palliative care, management of chronic medical deliverables, and psychosocial support for stable patients has become increasingly provided by nurses. We believe that the experience documented here serves as a useful approach for integrated, decentralized initial management and chronic care for oncologic disease at district hospitals. Regarding mentorship, the limited availability of oncologists in-country does create a need for ongoing clinical guidance from oncologists outside of Rwanda. Access to technical support on a near daily basis is critical to provide quality care in a task-shifted approach that largely depends on non-oncologists. The routine and broad support from Dana-Farber/Brigham & Women’s Cancer Center was critical to the implementation and growth of the cancer program. Lastly, outpatient oncology services in most rural settings require a high volume of referrals to higher levels of care for routine pathology, staging, chemotherapy, radiotherapy, and surgery. The BCCOE in the rural northern province of Rwanda has been well-documented as an exception given its broader spectrum of chemotherapy, pathology, and surgery services being readily available onsite at district hospital [10,22,24]. Outpatient oncology services in Rwinkwavu are ongoing, and the RMoH is exploring its applicability to other district hospitals in the country.

Costing analysis for this integrated clinic was not included in this study and should be a focus of future studies. The cost of care for outpatient services, including NCDs, is covered by the country’s national health insurance program, Mutuelle de Sante, whereby 10% of total costs are covered by the patient and the remainder by the government. Covered costs for district hospital outpatient services include common and severe NCDs, including type 1 diabetes and rheumatic heart disease. For health centers, common NCDs, such as type 2 diabetes and hypertension, are covered. However, for cancer services across all hospitals, services covered by Mutuelle de Sante are fewer, but growing. Intravenous chemotherapy remains heavily dependent on external funding at BCCOE. That said, since 2005, PIH/IMB has been committed to support the RMoH with the long-term plan of continuous transition of resource responsibility to the RMoH. As was done with the HIV program at RDH, this transition is ongoing for the integrated NCD clinic, including oncology services. As the breadth of NCD services and patient volumes grow, future studies will need to address the costs and sustainable financing strategies.

Our study does have several other limitations. We used routinely-collected programmatic data abstracted from the EMR, and some data points were incomplete or may have been inaccurate. Particularly, we had limited data regarding the stage at presentation and treatment pathways (e.g., curative versus palliative intent) for patients with malignancies. We suspect that a substantial fraction of patients with malignancies at first-level hospitals will initially present at an advanced stage [25]. The limited study window and restriction to newly diagnosed cases at only one site left us with a small sample size for many of our reported conditions. Additionally, we do not report extensive disease-specific outcomes, but this information has been published elsewhere for several of the disease groups in combination with the cohort from Butaro Cancer Center of Excellence [22,25,26,27,28,29,30,31]. An additional limitation of our study is that transfer data was not available in the EMR, so transferred patients may have been recorded as LTFU. Lastly, this study did not address quality of care or have a control group. As a result, detailed comparative analysis of the clinical outcomes was not possible as to evaluate the outcome of the novel approach. Based on information learned through this study, mitigation strategies for some of these limitations could include the modification and scale up of EMR systems in Rwanda. This could lead to routine monitoring and evaluation reporting for NCDs, which could drive quality improvement. Key quality-of-care metrics beyond clinical outcomes would include fidelity to clinical protocols (e.g., Are tests being ordered at the appropriate frequency?). The EMR should also expand its breadth of advanced NCDs covered, including chronic liver and kidney diseases. The RMoH is currently in the process of scaling the NCD EMR platform from RDH nationally.

This study further illustrates that the integrated approach, including the sharing of infrastructural resources across multiple diseases, can result in a feasible, routine NCD care delivery system at the district level. This integrated approach was essential to bypass the traditional, vertical approach which would not have been economically feasible in Rwinkwavu and many other rural, resource-limited districts. Furthermore, unlike referral level care, a district-based integrated approach allowed for NCD services to be geographically more accessible with all co-morbidities cared for in a single visit. This also results in adequate patient volumes. Lastly, the inclusion of basic oncology services within the integrated platform does require additional implementation steps unique to the complexities of cancer care. Leveraging on the integrated approach allows for such specialized services to be readily delivered from both a resource and operational perspective. Further quantitative and qualitative research surrounding patient outcomes and its drivers, particularly with respect to LTFU, and scalability would be of value to this population.

## Conclusions

We have documented an experience with integrated care for severe chronic NCDs, including malignancies at a first-level hospital in rural Rwanda. Short-term patient retention was high with this service delivery approach. We believe that this approach can be replicated in other district hospitals in low-income, SSA countries, and offers a pathway to universal health coverage with equity in this region.
